# Are We Ready for Migrastatics?

**DOI:** 10.3390/cells10081845

**Published:** 2021-07-21

**Authors:** Jonathan Solomon, Magdalena Raškova, Daniel Rösel, Jan Brábek, Hava Gil-Henn

**Affiliations:** 1 Cell Migration and Invasion Laboratory, The Azrieli Faculty of Medicine, Bar-Ilan University, Safed 1311502, Israel; yhontn@gmail.com; 2 Laboratory of Cancer Cell Invasion, Department of Cell Biology, Charles University, Viničná 7, 128 44 Prague, Czech Republic; magda.raska@gmail.com (M.R.); rosel@natur.cuni.cz (D.R.); 3 Biotechnology and Biomedicine Center of the Academy of Sciences and Charles University (BIOCEV), Faculties of Charles University, Průmyslová 595, 252 42 Vestec u Prahy, Czech Republic

**Keywords:** cancer metastasis, clinical trials, metastasis-free survival, metastasis inhibitor, research and development

## Abstract

Metastasis accounts for the highest mortality rates in solid tumor cancer patients. However, research and development have neglected this most lethal characteristic and, instead, have concentrated on the hallmarks of cancer that make tumor cells highly proliferative and distinctive from nonmalignant cells. The concentration on invasion and metastasis can be one of the most meaningful advancements in cancer investigation. Importantly, metastasis-free survival (MFS) was recently approved by the Food and Drug Administration (FDA) as a novel primary endpoint in clinical trials and has been used to evaluate the prognosis of patients with nonmetastatic castration-resistant prostate cancer and soft tissue sarcoma. This new definition enables to shift the focus of research and development in cancer therapeutics toward metastasis and to change the emphasis from using tumor shrinkage as a benchmark for indicating the efficacy of treatment to using MFS as a more representative endpoint for antimetastatic drugs. This perspective outlines the possibility to use this novel endpoint in other solid cancers, and examples of large clinical trials are given in which MFS is defined as an endpoint and/or in which antimetastatic strategies are being examined. These advances now open the door for the rapid development of antimetastatic therapies, which could be used in combination with standard cytotoxic cancer therapies. With pioneer research on metastasis prevention on the rise and the underlying biomechanisms of tumor cell motility and invasion explored further than ever before, we believe an intensified focus on antimetastatic properties will shape this era of cancer translational research.

Despite nearly two decades since the first six hallmarks of cancer were initially defined, therapeutic strategies have not given enough consideration to the mechanism that accounts for the highest mortality in solid tumor cancer patients: metastasis [1,2]. As a result, research and development in cancer treatments have not focused on malignant cells, the migratory capabilities of malignant cells, but, rather, on the hallmarks that make cancer cells highly proliferative and distinctive from nonmalignant cells [3]. The regression of solid tumors is conventionally described by the extent to which a treatment is antiproliferative and its subsequent ability to shrink the primary tumor. The current treatment approach uses the precise targeting of mutations and altered signaling pathways and proteins to slow or halt abnormal cellular proliferation. However, such an approach can be equated to shooting a moving target [4]. Treatments that target cellular viability and proliferation inevitably create an intense selective pressure on cells to acquire various mutations and, over time, to become resistant to antiproliferative therapies. Additionally, the current definition does not account for the potential of cellular migration and metastasis, thus rendering the regression criterion unreliable [4]. Instead of only focusing on slowing the proliferation of cancer cells, efforts should refocus on the deadliest characteristic of all malignant cancers: metastasis [3]. It is unsettling that, to this end, there are no antimetastatic drugs available to patients with solid tumors.

Metastasis is a complex sequential process in which primary tumor cells initially migrate through and invade the surrounding extracellular matrix. During dissemination, cancer cells invade the extracellular matrix (ECM) most commonly in clusters or sheets referred to as a collective migration, which requires proteolytic degradation at the leading edge and cell contractility in the following cells. Alternatively, cancer cells can detach and invade as individual cells, utilizing the protease-dependent mesenchymal migration modes or protease-independent amoeboid migration or a combination of both. What is more, many cancer cells can actively switch among these invasion modes in response to changes in the surrounding environment and/or to escape therapeutic intervention (for review, see reference [5]).

Following the initial invasion from the tumor, cancer cells usually enter the bloodstream and lymphatic system that transport them to new tissues. The cells that survive this rigorous process will invade a new tissue and establish a secondary tumor [3]. Antimetastatic drugs should not necessarily shrink tumors but can prevent the tumor cells from disseminating into the surrounding tissues. An additional advantage to this treatment strategy, which does not directly target cellular viability, is the low selective pressure and the absence of the proliferative advantage of cells resistant to antimetastatic drugs. It is also important to emphasize that antiproliferative therapies should complement antimetastatic treatments to be maximally effective.

Since the assumption of an antimetastatic approach as a promising therapeutic strategy, regulations evaluating the treatment efficacy have been appropriately adjusted. The 2018 draft guidance from the Food and Drug Administration (FDA) on the treatment of solid tumors defined the metastasis-free survival (MFS) of patients as a valid endpoint in clinical trials [6]. The guidance draft was motivated by robust results from three independent clinical trials that examined the effects of novel androgen deprivation therapies on preventing metastasis in nonmetastatic castration-resistant prostate cancer patients (nmCRPC) [1]. This draft is crucial to the future of clinical oncology for two reasons: First, regulatory mandated clinical trial endpoints are vital in steering the research and development focus and its investments towards metastasis [1]. Second, the draft guidance shifts the emphasis from using tumor shrinkage as a benchmark for indicating the efficacy of treatments to using MFS as a more representative endpoint for antimetastatic treatments. These two conclusions call for the rapid development of antimetastatic therapies.

Recent publications further support the redirection of the research and development focus on MFS as an accurate evaluative measure for cancer therapies. Of significance is the correlation of MFS with the overall survival (OS) of patients with nmCRPC [7]. It has recently been shown that metastasis development is associated with a significantly greater risk of death in men with high-risk nmCRPC; hence, MFS is predictive of OS [7]. When using MFS instead of OS as a clinical trial endpoint, investigators and clinicians will be able to observe the prognostic criteria earlier, allowing a more rapid evaluation of new anti-metastasis therapies.

Besides its use in clinical trials with prostate cancer patients, the value of perioperative therapy in soft tissue sarcoma patients will also be evaluated using MFS as a novel primary endpoint and indicator of OS. CHIC is a parallel, randomized, open-label, multicenter study assessing the effect of adding perioperative chemotherapy (as a standard of care) on MFS in patients with grades 1 to 2 soft tissue sarcoma. In this target selection design, 600 patients will be screened with a Complexity Index in SARcoma (CINSARC) to randomize 250 high-risk CINSARC patients between the standard of care and standard of care plus chemotherapy (four cycles of 3 weeks of intravenous chemotherapy with doxorubicin in combination with dacarbazine or ifosfamide, according to the histologic subtype). According to the investigators, low-risk CINSARC patients will be treated by the standard of care. The primary endpoint is MFS [8].

Another drug that is currently being explored as an antimetastatic inhibitor is rebastinib. Rebastinib is a monoclonal antibody that binds to Tie2, a protein receptor that promotes angiogenesis and, thus, is used as an antiangiogenic drug. In addition to this known property, rebastinib has shown some promise as an effective metastasis-targeting therapeutic agent in recent trials. Rebastinib is a Tie2-selective inhibitor used as an antiangiogenic drug and shows promise as an effective metastasis-targeting therapeutic agent. Several preclinical trials with mouse models of spontaneous breast carcinoma and pancreatic neuroendocrine carcinoma were conducted to reduce tumor intravasation and circulating tumor cells [9].

Additionally, the combination of rebastinib and paclitaxel increases the overall survival rate of paclitaxel-treated mice compared with mice treated with chemotherapy alone, even after resection of the primary tumor. Moreover, an observation using breast cancer patient-derived xenografts showed that rebastinib successfully inhibits the microenvironment-mediated intravasation of tumor cells [10]. Considering these results, rebastinib is now approved for several clinical trials [11]. According to clinicaltrials.gov, accessed on 20 July 2021, there are currently three ongoing trials that are testing the use of rebastinib alongside cytotoxic drugs to treat metastatic cancer [12,13,14]. These are phase I trials aimed at checking the safety of the treatment. Measurements for the overall effectiveness of the therapy (progression-free survival, time to progression, and OS) will also be taken.

A different therapeutic approach currently undergoing phase II clinical trials suggests targeting stress inflammatory mediators, such as catecholamines (adrenaline) and prostaglandins, due to their pro-metastatic influence. These studies use Propranolol, a β-adrenolytic agent, and Etodolac, a nonsteroidal anti-inflammatory drug, to suppress these inflammatory mediators to reduce the risk of postoperative metastasis in colorectal cancer and pancreatic cancer [15,16]. This trial was preceded by animal studies that showed efficient preventive abilities when both drugs were administered together, albeit with no effect when given separately. Later, two clinical trials were conducted in colorectal cancer patients and breast cancer patients. These trials showed favorable results in molecular and biological studies. An examination of the excised primary tumors showed a decreased capacity to migrate, a reduced pro-metastatic capacity of the malignant tissue, and improved immune infiltration. As the treatment aims to negate the stress and inflammatory response characteristic of the perioperative period, the administration should begin five days before the operation and then proceed for 20 days. In addition to measuring the overall success and tolerance for the treatment, the research will include mRNA profiling on the excised tissues. These will be taken to screen for molecular biomarkers related to metastatic and immune processes.

Further understanding of the physiological mechanisms related to metastasis can be achieved by observing the tumor microenvironment. A cohort model observational study estimated to end by 2026 is investigating the differences in the liver microenvironment between individuals and their role in liver metastasis, colorectal carcinoma, and pancreatic ductal carcinoma [17]. Tissue samples from liver metastasis and the primary tumors will be compared with samples from healthy volunteers, which will serve as a negative control. The primary aim is to identify tumor-associated antigens, regulatory pathways, and functional biological parameters and better describe the effects of therapies on antitumor immune responses. Tissue markers include the tumor microenvironment of metastasis (TMEM) density and mammalian-enabled (MENA) isoform expression patterns (Mena^Calc^ and Mena^INV^). TMEM is a lasting gateway for tumor cells to invade the bloodstream, and the density of TMEMs can be correlated with the likelihood of a tumor to metastasize [11]. The TMEM density can be visualized in the tissue samples by immunohistochemistry. MENA, an actin regulatory protein, is upregulated in tumor cells involved in the assembly of TMEM gates [18] and is highly involved in cell migration [19,20], as well as in the formation of invadopodia [21]. Mena^Calc^ and Mena^INV^ can be measured by quantitative immunohistochemistry or by qRT-PCR.

Understanding the tumor microenvironment could also lead to novel investigative techniques. A new study named TMEM-MRI (tumor microenvironment of metastasis-magnetic resonance imaging) aims to determine the ability and feasibility of new imaging technology. TMEM-MRI is performed on patients with confirmed or highly suspected invasive carcinoma and then compared with tissue sample findings to determine the accuracy of the imaging test [22]. Should TMEM-MRI prove valid, it is suggested that clinical practice can identify high-perfusion areas in tumors and estimate the cancer recurrence chances after treatment. Additionally, it can potentially assess the responses to preoperative therapy over time. A summary of the ongoing clinical trials in which MFS is defined as an endpoint and/or in which anti-metastasis treatments are examined is described in Table 1.

We commend these recent developments for redirecting cancer research and development efforts toward metastasis prevention. Since this strategy has yet to be explored extensively, additional clinical trials on other types of cancers should be conducted, with MFS as a novel primary endpoint. The use of MFS in new clinical trials represents a shift in our perception of solid cancer progression. The tumor size is no longer universally thought to be a reliable indicator for cancer progression and, most notably, its spread. The understanding of metastasis as an independent process that is not directly related to cell proliferation should prioritize new forms of treatment.

The conclusions from these studies will determine if MFS could be used as an indicator of prognosis in cancers other than nmCRPC. Furthermore, using MFS as a primary endpoint will propagate the study of cancer cell invasion as a critical process in cancer growth and, further, open doors to new avenues of investigation. For example, there is a great need to develop antimetastatic drugs that can be either newly developed or come from the current pharmacological therapies that show potential for repurposing as antimetastatic agents.

Finally, the term migrastatics, the identification and development of drugs that inhibit all modes of cancer cell motility and invasion, lends itself as an umbrella concept for future research and development in this growing wing of cancer research and clinical oncology [5]. With new regulatory pathways open and initial studies underway, it is the ideal time to enter into a new era of cancer translational research.

## Figures and Tables

**Table 1:** Ongoing clinical trials in cancer metastasis as obtained by ClinicalTrials (https://clinicaltrials.gov, accessed on 15 November 2020). The search queries used to retrieve the information on clinical trials in metastasis were: All studies, “Metastasis prevention”, “Metastasis inhibition”, “Metastatic microenvironment”, “Metastasis survival”, and “Metastasis prophylaxis” as the condition/disease. The results were manually checked for relevance.

Name	Cancer	Period	Stage	Location	Type	Phase	Significance	link
Interest of Peri Operative CHemotherapy In Patients With CINSARC High-risk Localized Grade 1 or 2 Soft Tissue Sarcoma (CHIC-STS01)	STS	2020–2025	Recruiting	France	interventional	3	A new treatment application of chemotherapy using MFS as an evaluative measure	https://clinicaltrials.gov/ct2/show/NCT04307277 (accessed on 15 November 2020)
A Study of Rebastinib (DCC-2036) in Combination With Carboplatin in Patients With Advanced or Metastatic Solid Tumors	Breast/Ovarian/Others	2019–2022	Recruiting	US	Interventional	1	Evaluates the capacity of a new drug with suspected migrastatic properties to target migratory processes directly	https://clinicaltrials.gov/ct2/show/NCT03717415 (accessed on 15 November 2020)
A Phase 1b/2 Study of Rebastinib (DCC-2036) in Combination With Paclitaxel in Patients With Advanced or Metastatic Solid Tumors	Breast/Ovarian/Others	2018–2021	Recruiting	US	Interventional	1	Evaluates the capacity of a new drug with suspected migrastatic properties to target migratory processes directly	https://clinicaltrials.gov/ct2/show/NCT03601897 (accessed on 15 November 2020)
Phase Ib Study of Rebastinib Plus Antitubulin Therapy With Paclitaxel or Eribulin in Patients With Metastatic Breast Cancer	Breast	2016–2021	Recruiting	US	Interventional	1	Evaluates the capacity of a new drug with suspected migrastatic properties to target migratory processes directly	https://clinicaltrials.gov/ct2/show/NCT02824575 (accessed on 15 November 2020)
TMEM-MRI: A Pilot Feasibility Study of Magnetic Resonance Imaging for Imaging of TMEM (Tumor Microenvironment of Metastasis) in Patients With Operable Breast Cancer	Breast	2018–2022	Recruiting	US	Interventional	N/A	New imaging technology which attempts to detect metastatic potential by direct observation	https://clinicaltrials.gov/ct2/show/NCT03694756 (accessed on 15 November 2020)
Safety and Efficacy Study of Enzalutamide in Patients With Nonmetastatic Castration-Resistant Prostate Cancer (PROSPER)	Prostate	2013–2023	Active, not recruiting	US	Interventional	3	New hormone-deprevation therapy using MFS as a clinical trial endpoint	https://clinicaltrials.gov/ct2/show/NCT02003924 (accessed on 15 November 2020)
A Study of Apalutamide (ARN-509) in Men With Non-Metastatic Castration-Resistant Prostate Cancer (SPARTAN)	Prostate	2016–2022	Active, not recruiting	US	Interventional	3	New hormone-deprevation therapy using MFS as a clinical trial endpoint	https://clinicaltrials.gov/ct2/show/NCT01946204 (accessed on 15 November 2020)
Efficacy and Safety Study of Darolutamide (ODM-201) in Men With High-risk Nonmetastatic Castration-resistant Prostate Cancer (ARAMIS)	Prostate	2014–2021	Active, not recruiting	US	Interventional	3	New hormone-deprevation therapy using MFS as a clinical trial endpoint	https://clinicaltrials.gov/ct2/show/NCT02200614 (accessed on 15 November 2020)
Colorectal Metastasis Prevention International Trial 2 (COMPIT-2)	CRC	2019–2027	Recruiting	Israel	Interventional	2	Metastasis suppression by indirect means, with analysis of the microenvironment	https://clinicaltrials.gov/ct2/show/NCT03919461 (accessed on 15 November 2020)
Perioperative Intervention to Reduce Metastatic Processes in Pancreatic Cancer Patients Undergoing Curative Surgery (BC-PC)	Pancreatic	2019–2026	Recruiting	Israel	Interventional	2	Metastasis suppression by indirect means, with analysis of the microenvironment	https://clinicaltrials.gov/ct2/show/NCT03838029 (accessed on 15 November 2020)
Advanced Immune Gene and Cell Therapies for Liver Metastases	CRC/PDAC	2020–2026	Recruiting	Italy	Observational	N/A	The study of the liver’s microenvironment and molecular properties, and their effect on metastasis	https://clinicaltrials.gov/ct2/show/NCT04622423 (accessed on 15 November 2020)

## Data Availability

Not applicable.
